# Supplementary material for: The nutritional quality of cereals varies geospatially in Ethiopia and Malawi
Source: Nature. 2021 May 19;594(7861):71–6. doi: 10.1038/s41586-021-03559-3 (PMC8172382; doi:10.1038/s41586-021-03559-3)
Supplement: Supplementary file 1 — Reporting Summary [file 41586_2021_3559_MOESM1_ESM.pdf]

nature research

Corresponding author(s): DBPR: 2020-08-15287

Last updated by author(s): Sep 7, 2020

Reporting Summary

Nature Research wishes to improve the reproducibility of the work that we publish. This form provides structure for consistency and transparency in reporting. For further information on Nature Research policies, see our Editorial Policies and the Editorial Policy Checklist.

Statistics

For all statistical analyses, confirm that the following items are present in the figure legend, table legend, main text, or Methods section.

n/a	Confirmed	
☐	☒	The exact sample size (n) for each experimental group/condition, given as a discrete number and unit of measurement
☐	☒	A statement on whether measurements were taken from distinct samples or whether the same sample was measured repeatedly
☐	☒	The statistical test(s) used AND whether they are one- or two-sided<br>*Only common tests should be described solely by name; describe more complex techniques in the Methods section.*
☒	☐	A description of all covariates tested
☐	☒	A description of any assumptions or corrections, such as tests of normality and adjustment for multiple comparisons
☐	☒	A full description of the statistical parameters including central tendency (e.g. means) or other basic estimates (e.g. regression coefficient) AND variation (e.g. standard deviation) or associated estimates of uncertainty (e.g. confidence intervals)
☒	☐	For null hypothesis testing, the test statistic (e.g. F, t, r) with confidence intervals, effect sizes, degrees of freedom and P value noted<br>*Give P values as exact values whenever suitable.*
☒	☐	For Bayesian analysis, information on the choice of priors and Markov chain Monte Carlo settings
☐	☒	For hierarchical and complex designs, identification of the appropriate level for tests and full reporting of outcomes
☒	☐	Estimates of effect sizes (e.g. Cohen's d, Pearson's r), indicating how they were calculated

Our web collection on statistics for biologists contains articles on many of the points above.

Software and code

Policy information about availability of computer code

Data collection	Ref. 44: AfSIS. New cropland and rural settlement maps of Africa. Accessed January 10 2020 from http://africasoils.net/2015/06/07/new-cropland-and-rural-settlement-maps-of-africa/ (2015).<br>Ref 45: Walsh, M. & Wu, W. GeoSurvey data prediction workflows. Open Science Framework (OSF) Repository, https://doi.org/10.17605/OSF.IO/VXC97 (2020).<br>Ref. 46: ESA. Land Cover CCI data [Version 2.0.7, Land Cover data for 2015]. European Space Agency Climate Change Initiative. Accessed November 21 2017 from http://maps.elie.ucl.ac.be/CCI/viewer/download.php (2017).<br>Ref. 47: R Core Team. R: A Language and Environment for Statistical Computing. (R Foundation for Statistical Computing, Vienna, Austria, 2017).<br>Ref 48: Grafström, A. and Lisic, J. BalancedSampling: Balanced and Spatially Balanced Sampling. R Package Version 1.5.2, https://CRAN.R-project.org/package=BalancedSampling (2016).
Data analysis	Ref. 47: R Core Team. R: A Language and Environment for Statistical Computing. (R Foundation for Statistical Computing, Vienna, Austria, 2017).<br>Ref. 60: Hijmans, R. J. Geosphere: Spherical Trigonometry. R Package Version 1.5-7, https://CRAN.R-project.org/package=geosphere (2017).

For manuscripts utilizing custom algorithms or software that are central to the research but not yet described in published literature, software must be made available to editors and reviewers. We strongly encourage code deposition in a community repository (e.g. GitHub). See the Nature Research guidelines for submitting code & software for further information.

Data

Policy information about availability of data

All manuscripts must include a data availability statement. This statement should provide the following information, where applicable:

- Accession codes, unique identifiers, or web links for publicly available datasets
- A list of figures that have associated raw data
- A description of any restrictions on data availability

All data and code are freely available from the corresponding author (MRB), and available online at [GitHub link to be provided shortly].

Field-specific reporting

Please select the one below that is the best fit for your research. If you are not sure, read the appropriate sections before making your selection.

☐ Life sciences ☐ Behavioural & social sciences ☒ Ecological, evolutionary & environmental sciences

For a reference copy of the document with all sections, see nature.com/documents/nr-reporting-summary-flat.pdf

Ecological, evolutionary & environmental sciences study design

All studies must disclose on these points even when the disclosure is negative.

Study description

Sampling and analysis of cereal grains from farmers' fields (standing mature crops) and local cereal stores.

Research sample

The research sample represents a composited cereal grain sample, together with a co-located soil sample, collected from a standard support frame (see below). Grain samples reported from Ethiopia were from teff (Eragrostis tef (Zucc.) Trotter; n=373), wheat (Triticum aestivum L.; n=328), maize (Zea maize L.; n=302), sorghum (Sorghum bicolor (L.) Moench; n=138), barley (Hordeum vulgare L.; n=181) and finger millet (Eleusine coracana (L.) Gaertn.; n=39), with a smaller number of Triticale (× Triticosecale Wittm. ex A. Camus; n=20) and rice (Oryza sativa L.; n=8) samples. Grain samples reported from Malawi were mostly maize (n=1,608), with sorghum (n=119), rice (n=54), pearl millet (Pennisetum glaucum (L.) R.Br.; n=31), and a single finger millet sample.

Sampling strategy

The objective of this study was to support spatial mapping of grain calcium (Ca), iron (Fe), selenium (Se) and zinc (Zn) concentration of cereal crops, across the largest areas of arable cropland in Ethiopia and Malawi that was feasible to sample.

The sampling strategy used “main-site” and “close-pair” sampling to support estimation of parameters of the spatial Linear Mixed Model (LMM). In Ethiopia (Amhara, Oromia and Tigray Regions), target sample frames were constrained to locations at which the probability of the land being under crop production was ≥0.9 based on predictions produced on a 500-m grid. These predictions were derived from the interpretation of high-resolution satellite imagery by trained observers and by machine learning methods applied to multiple covariates derived from remote sensor data and digital elevation models [Refs. 44,45]. The sample frame was further constrained to include only those locations from a 250-m grid, that fell within 2.5 km of a known road. A map indicating nodes on a 250-m grid (with the same origin as the agricultural land use grid) which met this requirement was prepared. These constraints may introduce possible biases into predictions made at locations outside the designed sample frame, however, it would not otherwise have been possible to visit all of the sample locations across in the time available. Information on the distribution of roads was taken from OpenStreetMap (www.openstreetmap.org). In Malawi, the cropland area was determined from the European Space Agency Climate Change Initiative [Ref. 46]. The agricultural area used was defined as including all raster cells which included the category of ‘cropland’ in their description. In Malawi, where road access is generally better to cropped areas than in Ethiopia, no constraint to road distance was imposed on sample locations. The mapped cropland areas are shown in Figure 1.

In Ethiopia, a total of 1,825 primary sample locations were selected a priori, with each 250-m grid node within the sampling frame allocated an equal prior inclusion probability. This was done using the lcube package from the Balanced Sampling library for the R platform [Refs. 47,48]. This implements a cube method, to enable sampling according to specified inclusion probabilities while aiming for balance and spread with respect to specified covariates [Ref. 49]. Here, sample locations were selected for spatial balance, which entails that the mean coordinates of sample locations are close to the mean coordinates of all points in the sample frame, and spatial spread, which ensures that the observations are spread out rather than clustered with respect to spatial coordinates [Ref. 50]. A subset of 175 of these locations were selected as “close-pair” sites where an additional nearby sample would be taken to support estimation of parameters of the spatial LMM [Ref. 43].

In Malawi, a different sampling method was used to achieve good spatial coverage of a total of 1710 main-site locations. These included 820 fixed sample points from the 2015/16 Demographic and Health Survey of Malawi [Refs. 24,51]. The stratify function in the spcosa package for the R platform [Ref. 52] divides a sampling domain into Delaunay polygons centred on a set of fixed points and with the remaining polygon centroids selected to partition the domain into approximately equal-area regions. The centroids of the polygons were selected as sample points. An additional 890 sample points were found in addition to the 820 fixed ones, with the stratify function. Once these were obtained, a further 190 locations were selected at random as “close pair” sites for an additional nearby sample, as in Ethiopia.

Data collection

Sampling was conducted by teams who were trained in standard procedures and risk assessments. Each team planned to visit five main-site locations per day. Main-site locations were loaded onto a computer tablet and printed on paper maps for each team. A team would navigate to the target location, using a GPS for the last few kilometres. At each sample location, the team would identify the nearest field with a mature cereal crop within a 1-km radius, and sample grain and soil, subject to farmer consent. If a field with a standing mature cereal crop was not apparent, then the team would ask the farmer to identify a field from which a cereal crop had

recently been harvested and stored, and from which a sample could be obtained. If sampling was not possible, then the team would either look beyond a 1-km radius for an alternative location, or abandon the location. At designated close-pair locations, a second field was identified ideally within ~500 m (range 100–1000 m) of the main-site location. If a close-pair location could not be found, then a close-pair location would be selected at the next sample location not already earmarked for a close-pair sample.

Within a selected field, samples were taken from a 100 m2 (0.01 ha) circular plot. This was centred as close as practical to the middle of the field unless this area was unrepresentative due to disease or crop damage. Five sub-sample points were located (Extended Data Figure 1). The first point was at the centre of the plot. Two sub-sample points were then selected at locations on a line through the plot centre along the crop rows, and two more points on a line orthogonal to the first through the plot centre. Where possible, the central sampling location was fixed between crop rows, and the 'long' axis of the sample array (with sample locations at 5.64 and 4.89 m) was oriented in the direction of crop rows with the 'short axis' perpendicular to the crop rows. A single soil subsample was collected at each of the five sub-sample points with a Dutch auger with a flight of length 150 mm and diameter 50 mm. The auger was inserted vertically to the depth of one flight and the five sub-samples stored in a single bag. Where a mature/ripe crop was still standing in the field, grain samples were taken close to each augering position, by a different operator, to minimise further contamination by dust and soil. For maize, a single cob was taken at each of the five points. Maize kernels were stripped from ~50% of each cob lengthways and composited into a single sample envelope for each location. For smaller-grained crops, sufficient stalks were taken so that approximately 20-50% of the sample envelope was filled (dimensions 15 cm × 22cm), with samples placed grain-first into the sample bag and the stalks were twisted off the grain heads and discarded. If a crop was in field stacks, then a sub-sample, comprising five cobs for maize, or a representative sample for other crops was taken from each available stack, taking material from inside the stack to minimise contamination by dust and soil (Figure S2). If a crop was in a farmers' store, i.e. already averaged from across the field, then a representative sample was taken, whilst avoiding grain from the store floor if grain was loosely stored and avoiding grain with visible soil or dust contamination.

Photographs at sample locations and of sample bags were recorded for quality assurance along with site GPS locations. In Ethiopia, 1,385 of the 1,389 locations from where grain data are reported had positional uncertainties of ≤8 m as recorded by the GPS. The other four locations had positional uncertainties of 9–16 m. In Malawi, 1,786 of the 1,807 locations had positional uncertainties of ≤9 m. A further 16 locations had positional uncertainties of 10–17 m, whilst six locations had positional uncertainties of 2,900–5,000 m. We took a decision not to exclude any data based on positional uncertainties for this study. We used robust estimators of the variograms (Extended Data Figure 2) which are resistant to effects of spatial outliers due to a small number of points being in the wrong position [Ref. 53] and these models were validated. Any effect of position error on the broad mapped pattern of long-range spatial variation at national scale will therefore be very limited and localised.

Grain micronutrient analyses followed standard methods [Ref. 54e]; these were conducted in the laboratory by named co-authors of the paper (Mossa, Wilson).

Timing and spatial scale

Timing: Grain and soil sampling from farmers' fields were completed in November–December 2017 (for most of Amhara Region) and November 2018–January 2019 (Amhara, Oromia, and Tigray Regions) in Ethiopia, and in April–June 2018 in Malawi.

The sampling frame for Ethiopia represents ~354,000 km2 of cropland, which is most of the cereal production area in Ethiopia. The sampling frame for Malawi represents ~66,000 of km2 area of cropland. The sample locations are provided in Figure 1 of the main paper.

Data exclusions

No data were excluded based on positional uncertainties. We used robust estimators of the variograms (Extended Data Figure 2) which are resistant to effects of spatial outliers due to a small number of points being in the wrong position [Ref. 53] and these models were validated. Any effect of position error on the broad mapped pattern of long-range spatial variation at national scale will therefore be very limited and localised.

No adjustment was made for potential contamination of grain samples, e.g., with soil dust from the field or store using typical markers (e.g. Fe, vanadium). Two sorghum samples, taken from a grain store in Malawi, were excluded from the data analysis based on high concentrations of calcium, magnesium and other elements that were considered unlikely to have arisen from soil contamination. These exclusions are reported clearly in the manuscript.

Reproducibility

It would be possible to conduct repeated sampling at the same locations given the information reported.

Randomization

There were no treatment groups in this study; the sampling design described above embeds randomisation.

Blinding

Not applicable to this study.

Did the study involve field work? ☒ Yes ☐ No

Field work, collection and transport

Field conditions

The sampling was conducted over a period of several months

Location

Exact locations for each sample are provided as metadata

Access & import/export

The fieldwork was conducted under formal ethical approvals from the University of Nottingham, School of Sociology and Social Policy Research Ethics Committee (REC); BIO-1718-0004 and BIO-1819-001 for Ethiopia and Malawi, respectively. These REC approvals were recognised formally by the Directors of Research at Addis Ababa University (Ethiopia) and Lilongwe University of Agriculture and Natural Resources (Malawi), who also reviewed the study protocols [NB, there are no recognised ethics committee that would cover low-risk sampling of agricultural fields in Ethiopia and Malawi].

Dried grain samples and dried soil samples was exported from Ethiopia and Malawi to UK, in compliance with all national and international laws, and with sample ownership retained by the lead institution in the country of origin (managed by a collaboration

	agreement). Material was moved into the European Community under Commission Directive 2008/61/EC, and Defra plant health licence number 105352/206483/3 (held by University of Nottingham).
Disturbance	All field sampling was conducted on arable/cultivated fields, with minimal disturbance.

Reporting for specific materials, systems and methods

We require information from authors about some types of materials, experimental systems and methods used in many studies. Here, indicate whether each material, system or method listed is relevant to your study. If you are not sure if a list item applies to your research, read the appropriate section before selecting a response.

Materials & experimental systems

n/a	Involved in the study
☒	☐ Antibodies
☒	☐ Eukaryotic cell lines
☒	☐ Palaeontology and archaeology
☒	☐ Animals and other organisms
☒	☐ Human research participants
☒	☐ Clinical data
☒	☐ Dual use research of concern

Methods

n/a	Involved in the study
☒	☐ ChIP-seq
☒	☐ Flow cytometry
☒	☐ MRI-based neuroimaging
